# Cholesteatoma Complicated by Parapharyngeal Abscess That Occurred After Temporal Bone Fracture

**DOI:** 10.7759/cureus.57523

**Published:** 2024-04-03

**Authors:** Rokas Abaturovas, Tomas Jakstas, Andrius Talijūnas, Svajunas Balseris, Irina Arechvo

**Affiliations:** 1 Department of Otorhinolaryngology, Faculty of Medicine, Medical Academy, Vilnius University, Vilnius, LTU; 2 Department of Otorhinolaryngology, Republican Vilnius University Hospital, Vilnius, LTU; 3 Center of Oral and Maxillofacial Surgery, Vilnius University Hospital Zalgiris Clinic, Vilnius, LTU

**Keywords:** chronic otitis media, temporal bone fracture, peritubal route, cholesteatoma, parapharyngeal abscess

## Abstract

Parapharyngeal abscess as a cervical complication of chronic otitis media with cholesteatoma is extremely rare. We present the case of a patient with chronic otitis media and cholesteatoma who developed a parapharyngeal abscess following a blunt head trauma. A 65-year-old man with a history of recurrent right purulent otorrhea presented with symptoms of profuse purulent otorrhea, headache, hoarseness, and difficulty swallowing. Imaging revealed the presence of a right parapharyngeal abscess alongside a temporal bone fracture, suggesting a potential direct spreading route of aggressive chronic suppurative otitis media infection through the bone fracture defects to the parapharyngeal space. The patient underwent abscess drainage via a transcervical approach with simultaneous emergency radical mastoidectomy. Despite the development of septic shock with acute renal failure in the postoperative period, the patient made a full recovery.

## Introduction

Although the incidence of parapharyngeal space infection and subsequent abscess formation has drastically decreased in the modern antibiotic era, it remains a clinical challenge due to rapid progression and possible severe complications [1]. Notably, it appears as the second most common deep neck space infection, mostly secondary to contiguous spread from local sites, following retropharyngeal space infections in children and peritonsillar space abscesses overall [2,3].

Parapharyngeal space abscess as a cervical complication of chronic otitis media with cholesteatoma is extremely rare. To this date, only a few cases of this life-threatening complication have been reported in the literature [4-6]. The different routes of extension, through the eroded mastoid tip or due to the involvement of the apex of the petrous temporal bone, have been previously described [4,6]. However, the appropriate time and surgical strategies for managing the complication and principal disease are still controversial. We present the first case of chronic otitis media with cholesteatoma complicated by a parapharyngeal abscess following a temporal bone fracture. This case was previously presented as a meeting poster at the 10th International Conference on Cholesteatoma, held in Edinburgh, United Kingdom, on June 5-8, 2016.

## Case presentation

A 65-year-old man presented to the neurosurgery department of our tertiary care facility after blunt head trauma. The initial high-resolution computed tomographic (CT) scans of the head revealed several hyperdense signals in the midbrain and brainstem that were interpreted as hemorrhage. A fracture of the right temporal bone was diagnosed as well. The right mastoid cavity was filled with soft tissue density debris. The patient had a long history of recurrent right purulent otorrhea. Three years before, he was treated for the same side temporal bone fracture. The previous CT temporal bone scans were re-evaluated, and a diagnosis of large atticoantral cholesteatoma was obtained.

**Figure 1:**
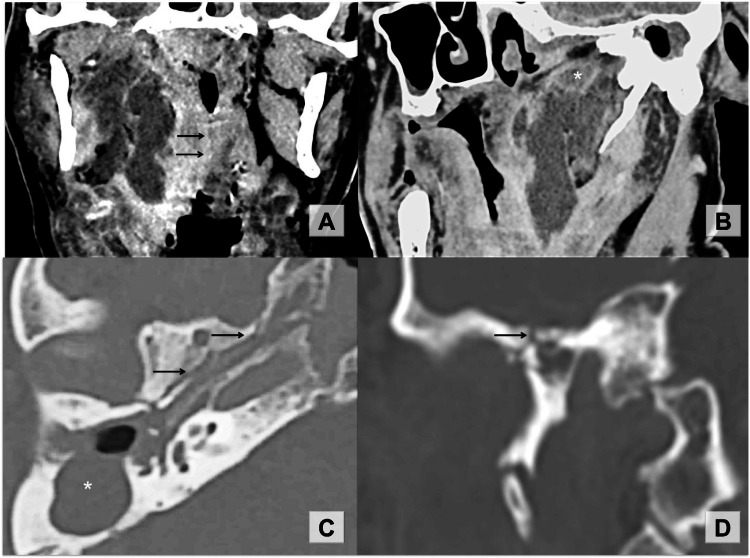
Temporal bone CT, contrast-enhanced scans (A) a multilocular parapharyngeal abscess appears as a lesion with a hypodense center and contrast-enhanced ring on the periphery. Edema and bulging of the pharyngeal wall are shown with the arrows. (B) Oblique sagittal reformat showing infiltration of the deep soft tissues of the skull base (asterisk), peritubal infection spreading route from the middle ear. (C) Oblique axial reformat along the whole peritubal fracture line (arrows). Large cholesteatoma destructed the posterior canal wall and filled the antrum as shown by an asterisk. (D) Comminuted fracture of the petrous paratubal part of the temporal bone (arrow), coronal CT scan.

In 12 days, the patient started to complain of profuse purulent otorrhea, headache on the right side, hoarseness, odynophagia, and dysphagia. On otoscopy, the external ear canal and the tympanic cavity were filled with pus and granulation tissue. Pharyngolaryngeal examination revealed edema of the soft palate and bulging of the lateral pharyngeal wall on the right side. Inflammatory markers were raised. Contrast-enhanced neck CT was performed, and a right parapharyngeal space abscess was diagnosed (Figures 1A, 1B).

Peritubal fracture lines (Figures 1C, 1D) of the temporal bone located in the center of the parapharyngeal space roof and the peritubal infiltration of the deep soft tissues of the external skull base surface (Figure 1B) suggest the direct spreading route of aggressive chronic suppurative otitis media infection to the right parapharyngeal space.

The general condition of the patient quickly deteriorated; he was diagnosed with septic shock and was admitted to the intensive care department. Urgent drainage of the parapharyngeal abscess through the external approach was carried out with simultaneous canal wall-down mastoidectomy without tympanoplasty.

Despite polyorganic insufficiency developed in the early postoperative period, the patient completely recovered. Three months after surgery, otologic examination revealed a dry mastoid cavity with completely dry tympanic membrane perforation. The patient did not report any otologic complaints.

## Discussion

Cholesteatoma-associated parapharyngeal abscess is rare, typically resulting from the infection spread through various anatomical pathways. For example, the erosion of the mastoid tip may facilitate the extension of infection along the digastric or sternocleidomastoid muscle reaching the parapharyngeal region. Although rare, infection of the apex of the petrous bone can proceed inferomedially to extend directly into the parapharyngeal space [4,6]. Other possible routes of infection spread should also be considered, such as the eustachian peritubal extension and suppuration of the lymph nodes along the upper part of the internal jugular vein, indicating lymphatic involvement [4-6]. Internal jugular vein thrombophlebitis secondary to the downward extension of lateral sinus and jugular bulb thrombophlebitis, which suggests vascular involvement, may also lead to the formation of parapharyngeal abscess [4,6].

**Figure 2:**
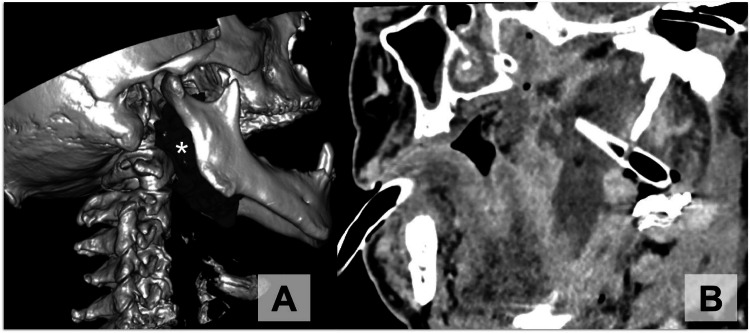
Temporal bone CT 3D reconstruction and drain position scans (A) The patient's temporal bone CT 3D volume-rendering reconstruction using the region-of-interest (ROI) technique. The total volume of 40 cm of the abscess (asterisk) was calculated preoperatively. (B) Oblique sagittal reformat of the patient's CT scan after surgical drainage showing the exact position of the drain in the abscess cavity.

The presented case shows that temporal bone fractures in patients with pre-existing chronic otitis media with cholesteatoma can cause infection spreading to the deep neck spaces through fracture defects, which were clearly delineated by high-resolution CT using oblique multiplanar reconstructions (Figure 2A).

Symptoms of parapharyngeal abscess may vary among patients but commonly include fever, sore throat, dysphagia, dysphonia, and trismus [2-4]. Clinical inspection may reveal signs such as oropharyngeal asymmetry (medial bulging of the lateral pharyngeal wall), tenderness, and torticollis of the neck due to spasm of the sternocleidomastoid muscle [2-4,6]. Additionally, induration and swelling below the angle of the mandible may be observed, along with the presence of lymphadenopathy [2,3]. In parapharyngeal abscess due to otitis media, otoscopic examination may reveal an inflamed or perforated eardrum, with visible pus or discharge in the external auditory canal. Notably, external signs of a deep neck abscess may be mild or absent, as seen in the presented case where only mild neck tenderness was noted preoperatively. Early recognition of possible complications is crucial; if a patient with chronic purulent otitis media develops symptoms like dysphonia and dysphagia, prompt consultation with an otologist is advised.

The parapharyngeal abscess should be differentiated from the congenital as well as benign and malignant lesions. It is important to note that neoplastic lesions displace the parapharyngeal fat tissue in predictable patterns [7]. In the presented case, the fat space was almost fully occupied by homogenous hypodensive mass with the contrast-enhanced ring. Perifocal cellulitis in the pharyngeal mucosal space appeared as homogeneous soft tissue swelling (Figure 1A). The imaging options for assessing parapharyngeal space region are limited to CT and magnetic resonance imaging (MRI) due to the challenge of obtaining a dependable sonographic view [1,2,7]. Despite that contrast-enhanced CT provides less information than gadolinium-enhanced MRI, this imaging technique was the only accessible modality in the emergency department.

As of today, surgical drainage is the treatment of choice for parapharyngeal abscesses [1,2]. It is worth mentioning that the three-dimensional processing of images (Figure 2A) can be performed by the surgeon immediately before an urgent operation in order to perform an external incision and to insert drains more precisely and less invasively (Figure 2B).

However, the appropriate time and surgical management of parapharyngeal abscess caused by chronic otitis media with cholesteatoma remains a topic of discussion. Rijuneeta et al. reported treating their patient with urgent transcervical incision followed by modified radical mastoidectomy 48 hours after admission to the hospital [4]. In our case, we performed a simultaneous neck incision and abscess drainage along with an emergency radical mastoidectomy. The rationale for the combined surgery was based on the patient’s severe foul-smelling otorrhea and significantly deteriorating septic condition. The additional risk of the radical mastoidectomy was thought to be negligible.

In retrospect, given the complex nature of the case and the rapid deterioration of the patient's condition, our experience underlines the importance of early diagnostic measures. Early CT scan analysis, with emphasis on identifying the continuation of the fracture line, could have potentially prevented the development of the parapharyngeal abscess or alleviated its severity.

## Conclusions

Temporal bone fracture in patients with pre-existing chronic otitis media with cholesteatoma can cause the infection to spread to the deep neck spaces through the fracture bone defects. Early consultation with an otologist is indicated in such instances. High-resolution imaging techniques, such as CT, play a vital role in identifying the extent of the infection and guiding surgical treatment. Although the surgical treatment strategy for parapharyngeal abscess as a complication of chronic otitis media with cholesteatoma remains debatable, we performed simultaneous neck incision and abscess drainage alongside emergency radical mastoidectomy.
